# Using common practices to establish a framework for mobile produce markets in the United States

**DOI:** 10.5304/jafscd.2021.104.029

**Published:** 2021-09-16

**Authors:** Christina M. Kasprzak, Julia J. Schoonover, Deanna Gallicchio, Lindsey Haynes-Maslow, Leah N. Vermont, Alice Ammerman, Samina Raja, Laurene Tumiel-Berhalter, Lucia A. Leone

**Affiliations:** a Department of Community Health and Health Behavior, School of Public Health and Health Professions, University at Buffalo; 329 Kimball Tower; Buffalo, NY 14214 USA;; b Department of Sociology, The College of Arts and Sciences, University at Buffalo;; c Department of Exercise and Nutrition Sciences, School of Public Health and Health Professionals, University at Buffalo;; d Agricultural and Human Sciences, North Carolina State University;; e Department of Community Health and Health Behavior, School of Public Health and Health Professions, University at Buffalo;; f Department of Nutrition, Gilling School of Public Health, University of North Carolina at Chapel Hill;; g Department of Urban and Regional Planning, School of Architecture and Planning, University at Buffalo;; h Department of Family Medicine, Jacobs School of Medicine and Biomedical Sciences, University at Buffalo;; i Department of Community Health and Health Behavior, School of Public Health and Health Professions, University at Buffalo;

**Keywords:** Diet, Food Access, Implementation, Public Health Practice, Mobile Market, Lower-Income

## Abstract

Access to affordable fruit and vegetables (F&V) remains a challenge within underserved communities across the United States. Mobile produce markets (mobile markets) are a well-accepted and effective strategy for increasing F&V consumption in these communities. Mobile market organizations share similar missions that focus on food, health, and empowerment, participate in incentive programs, offer nutrition education, utilize grassroots-based marketing strategies, prioritize local produce, and sell competitively priced produce through a market style. While mobile markets have become increasingly prevalent, models vary widely. Establishing standardized practices is essential for ensuring the effectiveness and sustainability of this important food access program. This research seeks to identify common practices of established mobile markets and describe the resources they rely on.

## Introduction

Effective, sustainable, and culturally acceptable interventions targeting underserved populations are needed to reduce disparities in dietary intake and decrease the prevalence of diet-related diseases such as heart disease, type 2 diabetes, obesity, and some cancers (Braveman et al., 2010). Fruit and vegetable (F&V) consumption is significantly lower in lower-income neighborhoods and communities of color and may be a contributing factor to disease risk (Grimm et al., 2012). Limited access to F&V has been identified as a barrier to consumption, spurring an emergence of research and strategies to increase access to healthy food (Haynes-Maslow et al., 2015; Haynes-Maslow et al., 2013; Kasprzak et al., 2020; Zenk et al., 2011). Mobile produce markets, or mobile markets, are small markets that travel to communities to distribute and sell F&V (Hsiao et al., 2019). To address growing health concerns, mobile markets have grown in number and popularity throughout the U.S. (Hsiao et al., 2019).

Research indicates mobile markets are a viable solution for improving the food environment through increased availability and access to F&V. There is growing evidence of their effectiveness in influencing F&V purchase and consumption (Hollis-Hansen et al., 2019; Hsiao et al., 2019). More rigorous and large-scale evaluations of mobile markets show an increase in F&V intake ranging from one-half to one cup per day (Gans et al., 2018; Leone et al., 2018). Among food access programs (e.g., community gardens, healthy corner stores, etc.), mobile markets are perceived favorably among lower-income communities if convenience and affordability are ensured (Haynes-Maslow et al., 2015; Kasprzak et al., 2020; Zepeda et al., 2014). However, vulnerable populations have expressed a limited awareness and understanding of mobile markets and a reluctance to trust new vendors due to concerns surrounding the organization’s motives and mission (Kasprzak et al., 2020; Zepeda et al., 2014). Therefore, ample and strategic community engagement should take place before establishing a market in a new community.

Organizations that start mobile markets, primarily nonprofit entities, avoid some business risks associated with “brick and mortar” stores and can quickly adapt to communities’ needs (Hollis-Hansen et al., 2019; Leone et al., 2019). However, many organizations face challenges with community and organizational sustainability (Zepeda & Reznickova, 2016). Although there is growing interest in evaluating the impact of mobile markets on health, little is known about operational mechanics and what set of practices maximize the likelihood of reaching the target population and sustainability. Identifying common practices will provide a precedent for new markets to follow, avoiding the trial-and-error process that established mobile markets have previously experienced. Furthermore, the adoption of a standard set of practices by mobile markets will allow researchers to investigate whether a well-run mobile market can create positive change and facilitate replication and comparison across communities. Identifying common practices among mobile markets also helps to further legitimize this food access strategy. For example, while some states have established their own criteria, there is a lack of clarity in how a mobile produce market is defined by the United States Department of Agriculture (USDA). Summarizing practices can provide a framework to understand mobile market operations, prompting federal agencies to establish an accepted definition and facilitate organizations’ participation in federal nutrition assistance programs (e.g., Supplemental Nutrition Assistance Program [SNAP], Women, Infants, and Children Program [WIC], Farmers Market Nutrition Program [FMNP]). Finally, raising awareness and understanding of mobile markets may encourage researchers, policy-makers, funders, and other stakeholders to recognize the importance of mobile food access programming.

Most extant mobile market-focused research has been conducted with customers, with few studies focusing on operators. Studies that have surveyed organizations looked broadly at mobile food vendors, including those that sell nonproduce items, to assess the food environment (Lucan et al., 2014) and the proximity of vendors to each other and their target population (Lucan et al., 2013). Only three studies have focused more narrowly on the processes of mobile produce markets. Robinson et al. (2016) conducted 11 in-depth interviews with representatives from a single mobile market in Syracuse, New York, and observed 16 market sites operated by two organizations. Weissman et al. (2020) surveyed 50 U.S. and Canadian mobile markets. Zepeda and Reznickova (2016) conducted case studies in six U.S. communities with mobile markets. Our study furthers this research by focusing on more established mobile markets that have operated for at least two full years and by asking a broader scope of questions (e.g., community engagement, successes). The goals of this research are to use in-depth interviews with mobile market operators to identify common practices of established mobile markets and work towards establishing a framework for mobile produce market operations. We also summarized operators’ perspectives on the resources that most contributed to their success.

## Methods

### Recruitment and Enrollment

In the spring of 2018, a database of mobile market organizations was created by identifying organizations through word-of-mouth, internet searches, and a mobile market listserv. An outreach email was then sent to potential key informants (KIs) to briefly explain the study and direct them to a screening survey on the study’s website. Potential KIs answered questions related to their organization’s structure, duration of operations, and interest in being interviewed. Those who completed the survey were contacted via email or phone to verify their interest in the study and that their mobile market was operating.

KIs were eligible if they worked with organizations operating a mobile market in the U.S. for at least two years, were interested in sharing information about their market, and could speak to its model and sustainability. Of the initial 60 organizations contacted, 27 completed the survey, and 19 were eligible and interested. Of the remaining 33 mobile markets that did not complete the survey, five were successfully reached. Of these five, three were either ineligible or not interested, and two enrolled. Phone interviews were scheduled with the staff member(s) most familiar with the history and operations of the mobile market. Organizations that did not meet the eligibility criteria were encouraged to participate in other research and networking opportunities. This study was approved by the University at Buffalo Institutional Review Board.

### Interview Process

Two researchers conducted semistructured phone interviews lasting up to 90 minutes between May and November 2018. The research team developed the interview guide, drawing on collective experience in operating and evaluating mobile markets and similar programs. The guide focused on market models; logistics and operations; community engagement and marketing strategies; staffing, nutrition education, and ancillary services; procurement and pricing; program impact and evaluation; and business and financial models. The majority (*n*=19) of KIs agreed to identify their organizations in the findings so that case studies could be developed, and the study team could facilitate networking and information sharing between organizations. KIs were compensated US$50 for each interview.

### Data Analysis

All interviews were recorded, transcribed, and checked for accuracy. Data analysis was completed using the qualitative software program ATLAS.ti version 8.0. Transcripts were divided between two graduate research assistants to code utilizing a codebook of themes informed by the interview guide. Reports were generated for all codes and summarized each theme (e.g., organizational structure) across all mobile market organizations. Memos were written to summarize each code report, and frequency distributions were calculated for specific themes.

## Results

Twenty-five KIs representing 21 mobile markets participated in interviews (four organizations had two participants). No KIs withdrew from the study. Table 1 includes mobile market organization characteristics. The KIs represented organizations from 16 states and 19 cities in the U.S. The majority (*n*=14) serve predominantly or exclusively urban areas, with the remaining serving a mixture of urban, rural, and suburban (*n*=5) or exclusively rural (*n*=2) areas.

### Organizational Structure

Table 1 indicates the organizational structure of the represented mobile markets. The majority (*n*=17) are managed by a parent agency with missions to build and strengthen resilient food systems, empower communities, address food insecurity, and reduce health disparities. Separate but complementary services are commonly offered. Organizations serve a similar target market described in many ways but generally recognized as high-need and lower-income. Funding is often from a combination of sources but is predominantly from federal and regional grants and, to a lesser degree, produce sales. Other common funding sources include corporate sponsorship, fee-for-service events, philanthropy and donations, or entities such as a city/municipality, parent organizations, or foundations.

Staff may work directly at the market or indirectly in administrative or coordinating roles, and many markets share staff with other programs run by the parent organization. The number of market staff and the split between paid full/part-time staff and unpaid volunteers are highly variable among organizations; there is less variability for direct market staff with a range of one to three paid and one to five volunteers (mostly seasonal workers). It is common for employees to be responsible for several tasks, including running the market, customer service, cashing out customers, managing inventory, driving the vehicle, etc.

### Market Operations

All organizations use a model that emulates a farmers market experience, selling produce per item and permitting choice by customers. The rationale behind adopting this model was to create a familiar retail experience that allows for the “dignity of choice.” However, some organizations (*n*=4) also offer a preset box program similar to a community supported agriculture program (CSA). Most organizations utilize one to two trucks, vans, or busses to transport produce to sites and set up the market on the vehicle’s perimeter or within the host site, with few organizations operating the market exclusively within the vehicle. Regardless of vehicle or setup, organizations may adapt to cold climates by moving indoors. It is common for organizations to retrofit their vehicles to meet their specific needs, although the types of upgrades vary (e.g., storage, generators, solar panels). Few organizations have refrigerated vehicles and therefore invest in stand-alone or retrofit refrigeration for the vehicle or operations hub (e.g., Cool-Bot system, coolers, refrigerators).

Most organizations operate their market for at least half the year, with some running year-round (*n*=8). The weekly market schedule ranges from two to six days, averaging four days. The number of weekly market stops ranges widely (3–75), but organizations typically operate a market from one to four hours, with two hours being optimal. However, KIs cautioned there is no “hard and fast rule” for scheduling as it is highly dependent on the host site, customer demand, climate, staffing, and vehicle availability.

Prices are often set informally based on trial-and-error and comparing prices to local retailers. Some organizations reported that they had more methodical pricing strategies in the past but then shifted to a more flexible approach that allowed them to respond to what customers are able and willing to pay, often in real-time. Several organizations sell produce close to or at the price they purchased it; when markups are used, they are applied within the range of 10–45% from the purchase price, with most falling in the 10–20% range. Most organizations perceive their pricing to be comparable to grocery stores and less than farmers markets, colloquially described as “somewhere in between a Walmart and a Whole Foods price” and “as low as possible.” To further increase the affordability of produce, all organizations accept SNAP/EBT. Nearly all organizations participate in at least one F&V incentive program, including SNAP matching programs, regional-specific healthy food incentive programs, and Seniors’ and Women, Infant, and Children (WIC) Farmers’ Market Nutrition Program (FMNP) benefits. Organizations often create incentives to cast a wider net of eligibility to include lower-income customers not receiving SNAP benefits but receiving other government assistance (e.g., Medicaid, disability). Organizations may render incentives at the point of sale (e.g., vouchers, discounts, reward cards) or distribute vouchers throughout the community (e.g., events, health fairs).

All organizations offer some form of nutrition education, with most utilizing partner organizations (e.g., Extension office, health clinic, nutrition students/interns) to offer education on a weekly or biweekly basis, typically at the market or within the host site. Education can take on many forms, such as mini-lessons or pop-up grocery store tours; however, cooking demonstrations and tastings are the most popular among customers. Education can also be informal through distributing materials (e.g., recipes and handouts), engaging in conversation on handling or preparing produce, and inviting community partners to table at the market.

### Site Selection and Agreement

The majority of market sites are created through partnerships with community organizations that are already serving lower-income communities. Other methods of identifying sites include familiarity of high-need and food-insecure areas, community demand, trial-and-error, utilizing a food environment map, and findings from past food access research. When choosing to partner with prospective community sites, all mobile market organizations prioritize need—meaning there must be a high density of lower-income and/or SNAP-eligible households in the vicinity. Common host partners, including sites with the largest and most consistent customer base, are included in Table 1; however, there was not complete agreement as to which sites are the busiest. For example, one KI described health clinics as busy, whereas another KI cited health clinics as slow. Another KI explained there is great variation between sites of the same type.

Most organizations screen potential sites by meeting with a point of contact and having an informal agreement, or mutual understanding, with host sites regarding expectations for operating the market. However, some organizations create a memorandum of understanding or a similar contract. Organizations typically assess if the site is a good fit in terms of physical requirements (e.g., parking, bathrooms), capacity (e.g., marketing efforts, outreach), and viability (e.g., target market reach, volume). An organization’s expectations for each host site are site-dependent, and organizations largely “meet them where they are.” Still, community outreach and marketing are primarily the responsibility of the mobile market organization or a shared responsibility with the host site.

### Procurement and Logistics

Produce is sourced from a combination of wholesalers, aggregators, produce auctions, direct farm procurement, an organization’s own farm, and donated produce. While the percentages from different sources shift with the seasons and conditions, organizations are predominantly sourcing directly from farms. Factors influencing sourcing decisions include customer preference, climate, geography, price, the capacity of internal farm operations, linkages to farmers, and the overarching mission of the organization and/or mobile market. Almost half of the organizations (*n*=9) engage in some form of farming that may serve as a partial source of produce. All organizations recognize the importance of sourcing locally to support local farmers and the economy. KIs emphasized the need to balance this priority with their mission to provide affordable, culturally relevant produce that matches customer preferences while remaining financially sustainable. Organizations first attempt sourcing “as local as possible,” and if the season or price does not permit this, they will opt to source regionally (within the state or neighboring states) and, if necessary, through a wholesaler or distributor. About a quarter of the organizations are exclusively or almost exclusively sourcing locally, loosely defined by KIs as 100 miles or less from their location.

Organizations often want to support sustainable farming practices and procure more organic produce, but the price makes this prohibitive, and there has not been strong customer demand. Alternatively, organizations try to source produce that is Good Agricultural Practices (GAP)–certified or is grown using low-spray, integrated pest management, or organic-like practices. Markets carry a wide variety of produce (8–50 varieties), but 22–25 is the average range. Fruit is the consistent favorite among customers. Organizations commonly sell nonproduce items (e.g., eggs, canned and dried goods). Those with refrigeration at the market may offer nonproduce perishables (e.g., yogurt, cheese, meat, fish). All organizations have access to dry and cold storage at their operations hub, nearby storage, or refrigerated vehicle(s).

### Marketing and Community Engagement

KIs highlighted the importance of laying a strong foundation before establishing a mobile market by engaging with the community early and often through community events or meetings (e.g., health fairs, neighborhood resident meetings), speaking engagements, and connecting with policy-makers. KIs emphasized that cultivating strong relationships and effective communication with host sites ensures market stops are viable and reach their target market. A small number of organizations have a community advisory board, and most are interested in forming or reviving one.

The most common marketing strategies employed by organizations include canvassing, flyers and signage, broadcast (e.g., TV, radio), print and social media, digital outreach (e.g., text messages, emailed newsletters), ad campaigns, direct mail, and the visual appeal of the market. Other common strategies include word-of-mouth, implementing a consistent market schedule, attending community events, and networking. Most KIs felt their organization is adequately reaching their target market but recommended persistence and patience for new markets given the time it takes to build trust and recognition at new sites.

### Sales, Data, and Evaluation

The majority of organizations track sales and forms of tender (e.g., incentives) with point-of-sale software (e.g., Square, Farmers Register), with few using handwritten ledgers. Some organizations collect non-sales data, such as participation in assistance programs (e.g., SNAP), customer demographics, and feedback, through online platforms, paper surveys, or rapid market assessment. Many of the organizations have gone through some form of formal evaluation, often as a condition of funding. Formal evaluations have been carried out internally or in partnership with an external organization, such as a local university, and have measured variables including purchasing, demographics, customer and stakeholder feedback, sustainability, perceptions and connectedness to one’s neighborhood, diet, and impact on the healthcare system. Less formal evaluations include self-assessments of market operations and collecting customer feedback. Table 2 contains quotes from KIs illustrating common practices for each theme.

### Operator Perspectives on Resources That Contribute to Success

**Figure 1:**
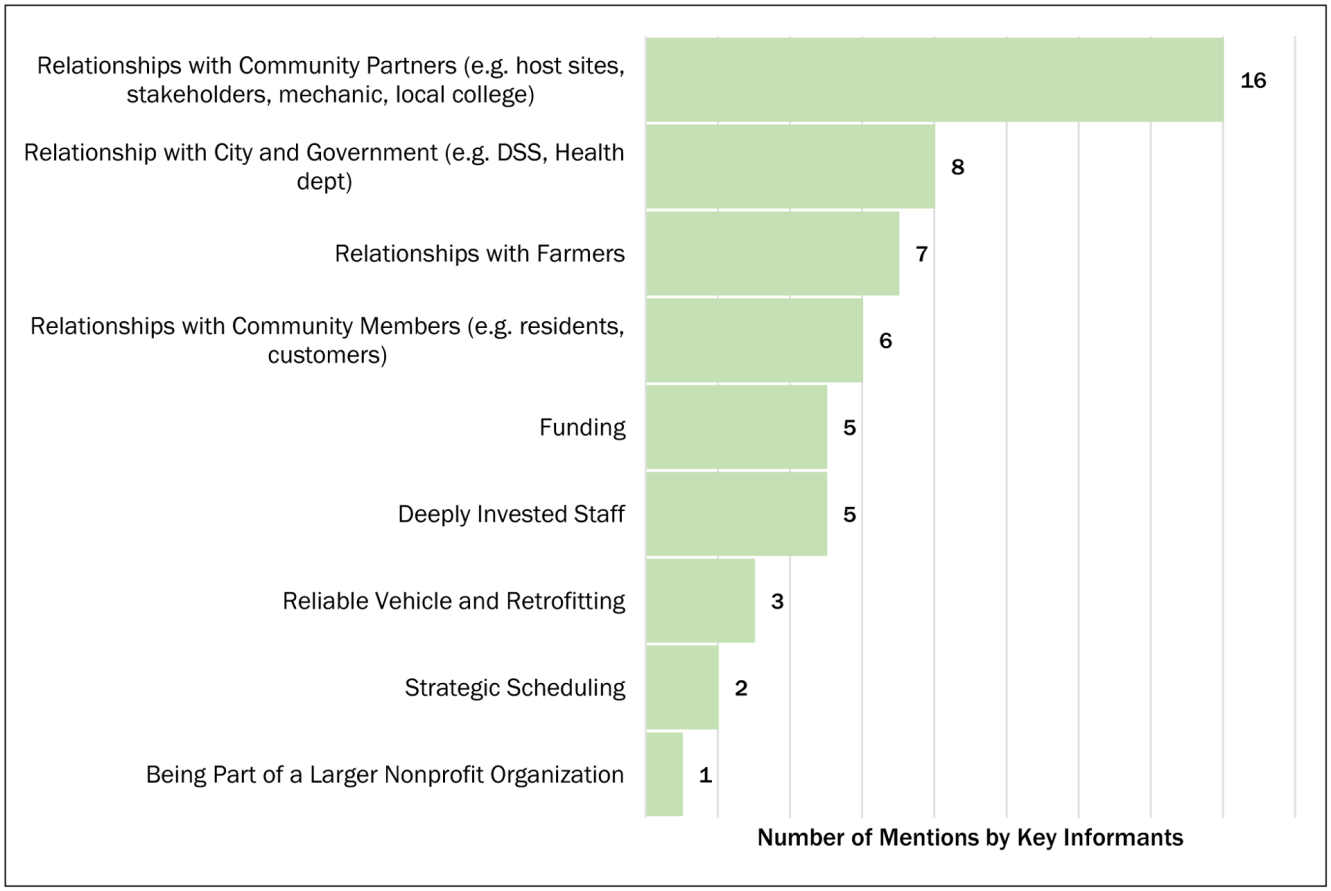
Resources That are Key to Mobile Market Success

In addition to common practices, KIs were asked which resources are key to the success of their mobile market. Figure 1 depicts the most cited resources. Relationships with partners, both grassroots and government, are paramount. Organizational features that contribute to success include sharing resources with a parent organization, hiring strong staff, and securing corporate sponsorship or grant funding. The viability of market sites was attributed to the strategic selection of host sites and optimized scheduling. A reliable vehicle that is customized to a market’s needs is also a valuable resource among organizations.

## Discussion

Interviews with KIs revealed several core tenets of mobile market practices that have informed our proposed framework for mobile produce markets. The following are key characteristics of mobile markets: (1) set up temporary food markets in partnership with organizations already serving the local community; (2) uphold an organizational mission to create equitable food access, bridge health disparities, and/or support local food systems; (3) operate a market model that permits customer choice; (4) sell produce and nonproduce items, prioritizing healthy, fresh food; (5) increase the affordability of F&V through pricing structure or incentive programs; (6) procure produce through multiple sources, but prioritize procurement of local and regional produce; (7) operate at least half the year; and (8) offer some type of food, nutrition, or cooking education. Areas with more variability in practices, which were excluded from the framework, include staff size and composition, vehicle type, specifics of funding and procurement sources, number of market stops, and scope and rigor of program evaluation. While most mobile markets primarily target lower-income individuals, we did not exclude markets that serve other demographics as we recognize that many markets serve multiple target populations, often using a sliding scale or cost-offset model to improve sustainability (Niebylski et al., 2015).

The findings of Robinson et al. (2016) align with the present study in terms of organizations’ stated missions and target markets, procurement practices, competitive pricing, acceptance of financial incentives, and the importance of community engagement and relationship building. The current study also supports findings from Weissman et al. (2020) that most mobile markets are managed by a nonprofit, serve a predominantly lower-income and low-access target market, and mainly rely on grassroots-based marketing. Weissman et al. (2020) similarly found most organizations prioritize nutrition education, participate in incentives programs, price produce competitively, have a wide number of market stops, operate for at least half of the year, offer nonproduce staples, and prioritize local procurement with organic produce being less of a priority. They also reported sales alone do not cover operational expenses, citing private foundation money as the main source of funding (Weissman et al., 2020), while the KIs interviewed in this study emphasized the importance of grant funding. The present study did not ask for percentages or dollar amounts of funding sources, which prevents a direct comparison to the findings of Weissman et al. (2020); nevertheless, the need to seek out additional funding sources and the variability between organizations is a shared finding. This study replicated many of these findings while providing additional details on practices that cannot be gained through quantitative studies.

The limitations of this research include the predominantly urban and Northeastern U.S. representation; therefore, these practices may not be generalizable to different geographies and communities. Not all KIs provided quantitative data for questions; therefore, numbers and averages supplied here do not represent all of the organizations interviewed. Given the exploratory nature of this research, we defined the success of a mobile market as longevity or the number of years operating. This broad eligibility criterion may not have adequately focused our attention on the most viable strategies. However, in the absence of an accepted definition of effectiveness for mobile markets and the scarcity of rigorous evaluations, we opted not to create eligibility criteria based on presumptions. Therefore, we are prudent in describing these practices as common practices rather than “best practices.” Lastly, our findings represent established mobile markets and may not wholly include models and practices of more nascent markets. Therefore, we anticipate that this framework will be dynamic and subject to revision and updates.

### Implications for Future Research and Practice

The present study furthers the research on mobile markets by helping to clarify common implementation practices and identifying effective, scalable, and ready models for broader adoption. A significant step toward standardization is the development of the Veggie Van (VV) Toolkit, a web-based collection of evidence-based practices to help organizations implement a mobile market following the VV model; the toolkit has been updated with these community-tested practices and made publicly available (Leone et al., 2018). Since the onset of the COVID-19 pandemic in the U.S., there has been a surge in new mobile markets; disseminating these practices in the toolkit reduces the burden on established organizations that are being solicited for guidance. These findings were also used to refine inclusion criteria for organizations that would participate in the VV study, an ongoing randomized controlled trial (RCT) evaluating the effectiveness and implementation of mobile markets. We also hope that our proposed framework will serve as an impetus for federal agencies, notably the USDA, to establish an accepted but flexible definition of mobile markets. In doing so, mobile markets will be recognized for their important role in the food system, addressing food insecurity, and ideally, streamlining policy processes that impact mobile market organizations.

Future research should continue to evaluate mobile market practices and create linkages with outcomes to further our understanding of how to ensure they are effective. Mobile markets have been deemed efficacious through evaluation in two RCTs on their impact on F&V consumption (Gans et al., 2018; Leone et al., 2018). However, F&V consumption is likely one of many outcomes that constitute researchers’ and practitioners’ notions of success. As such, we ought to understand how practitioners and community members define success and adjust our scope of research outcomes accordingly. Research is also needed to understand further how mobile market operations should be adapted to rural communities and how organizations have adapted their practices during the COVID-19 pandemic (e.g., pre-packed produce bundles). Lastly, the interviews in this study resulted in a significant amount of data beyond the common practices described here. We plan to report additional findings on common barriers experienced by mobile market organizations to highlight the support and resources needed to overcome persistent challenges.

## Figures and Tables

**Table 1.** Mobile Market Organization Characteristics

Region of the U.S.	Number of Mobile Market Organizations	Target Market	Percentage of Mobile Markets (n)
Northeast	10	Low to moderate-income individuals; demonstrating a need for food assistance (SNAP recipients)	86% (18)
South	6	Populations vulnerable to health disparities and chronic disease (seniors, housebound, racial and ethnic minorities)	76% (16)
West	3	Limited access communities (lack of fresh food, lack of transportation)	52% (11)
Midwest	2	–	–
Years Operating	Percentage of Mobile Markets (n)	Community Partners/Host Sites	Percentage of Mobile Markets (n)
3 years	19% (4)	Health care providers (clinics,^a^ VA medical center)	81% (17)
4 years	29% (6)	Community centers (general,^a^ senior,^a^ youth afterschool, YMCA)	81% (17)
5 years	19% (4)	Housing (low-income,^a^ transitional, assisted living)	57% (12)
6 years	5% (1)	Public institutions (libraries,^a^ primary and secondary education)	48% (10)
7 years	19% (4)	Public space (vacant lot, street parking, farmers market, community gardens)	24% (5)
8 years	5% (1)	Private companies (retail space, insurance company, law firm)	24% (5)
9 years	5% (1)	Government and social service providers (food pantry, WIC clinic,^a^ health departments, departments of social services, Head Start)	24% (5)
–	–	Faith-based organizations (church)	19% (4)
		^a^ Cited as a busier site	
Organizational Structure	Percentage of Mobile Markets (n)	Ancillary Services	Percentage of Mobile Markets (n)
Nonprofit (Other)	48% (10)	Education (gardening, nutrition, youth and leadership)	57% (12)
Nonprofit (Hunger Relief/Food Bank)	14% (3)	Agricultural activities (composting, vermiculture, urban and community farming)	52% (11)
Nonprofit (Hospital Network)	10% (2)	Public health programming (healthy corner stores, corporate wellness, Veggie Rx, farm to institution, SNAP matching)	38% (8)
Stand-alone Mobile Market Nonprofit	10% (2)	Produce sales (farmers markets, farm stands, CSA)	24% (5)
Nonprofit (Foundation)	5% (1)	Food aggregation and distribution (food hub)	14% (3)
Nonprofit (Public Health Entity)	5% (1)	Policy and advocacy work; coalition building	14% (3)
University/College	5% (1)	Emergency food assistance (food pantry, dining hall, meal and food box distribution)	14% (3)
City/Municipality	5% (1)	Professional development (job readiness training, internships, GED)	14% (3)
–	–	Public health promotion and outreach (SNAP enrollment, health screenings)	10% (2)
–	–	Foodservice (community kitchen, business incubator)	10% (2)
–	–	Community improvement (beautification, safety)	10% (2)
–	–	Social services programming (housing support)	10% (2)

**Table 2.** Common Practices Illustrative Quotes

Theme	Subtheme	Common Practices
Organizational Structure	Mission Statement	“Our [organization’s] mission is to build thriving communities through local food. But the mobile market mission is to directly improve the access to that local food.” “Our mission is to promote community leadership and create access to healthy food for our most food insecure communities.”
Market Operations	Market Set-up	“…Honestly, we totally cater to our shoppers. If they would request that we bring it inside, then we’ll bring it inside. Some of those locations, it’s actually gorgeous, of course, we’re going to set up outside. But again, we really cater to what they want and what they like because it’s just a matter of business.” “We set up outside the van, so it’s not a walk-on vehicle, the whole vehicle is filled with the produce. We had done some retrofits because we were thinking it might be a walk-on, but the volume of sales we do it’s not realistic for us. So it’s really they’re kind of popping up a farm stand everywhere we go. So, we have usually around four tables worth of produce.”
Host Site Selection	Screening and Agreement	“It’s been every year, and it’s still we’re still on a learning curve. I feel like I can figure it all out but what we really do is end up trying to identify strong community partners and areas of need and trying to develop relationships with businesses, nonprofits, property owners, whoever it may be, that we can identify as what we see as a successful stop.… We try and screen out for people who will and partners who [will] actually be engaged in helping us spread the word, whether that’s a nonprofit that views us as a service for their clientele or a neighbor or a neighborhood organization that really wants us to meet the need of their clients as well.“ “I wouldn’t say it’s like an MOU, but we do have the application they filled out, and it’s–we discuss like the terms that they need to be doing this outreach. And we do put in the application that they either need to meet our sales minimums or our visitor minimums.”
Procurement and Logistics	Produce Sourcing and Priorities	“During the growing season, we source from local farmers as much as we can, but it’s challenging because the cost of the food is higher with local farmers. So, what we’ve been doing is partnering with local farmers. We’ll take kind of their excess stuff that maybe isn’t, like their seconds and so they’re not that as good to sell…and then everything else is purchased wholesale.” “[During the growing season] we’re mostly local, and during the rest of the season, we’re probably down to about 10 percent local. The storage crops, apples, potatoes, onions, some squash, and then almost everything else is from wholesalers.”
Marketing and Community Engagement	Outreach Strategies	“We attend events, we drop off flyers, we do speaking engagements, like we do all sorts of stuff.… I do know, the door-to-door flyering is the best thing for us.” “I think a lot of it narrows down to community relationship. So, finding those allies in each neighborhood that we have, that are motivated and engaged and willing to spread the word for us. I mean it’s [the] same as marketing or product word of mouth is the most popular and it’s also the hardest to promote.”
Sales, Data, and Evaluation	Types of Data; Means of Data Collection	“I wouldn’t say like in a formal evaluation that we do like, season evaluation every winter, and like check, and ‘Hey, how did this work? How did that work? Let’s look at the numbers monthly, and like, are we on target for our transaction goals, are we on track for our average like, average market sales?“ “That was done through a partnership with a local university, so we’re using the systems that were developed in that first three food budget grants to do data collection.”
